# Supplementary figures and images for: Correlation of alpha-1 antitrypsin levels and exosome associated neutrophil elastase endothelial injury in subjects with SARS-CoV2 infection
Source: PLoS One. 2022 Sep 9;17(9):e0274427. doi: 10.1371/journal.pone.0274427 (PMC9462798; doi:10.1371/journal.pone.0274427)

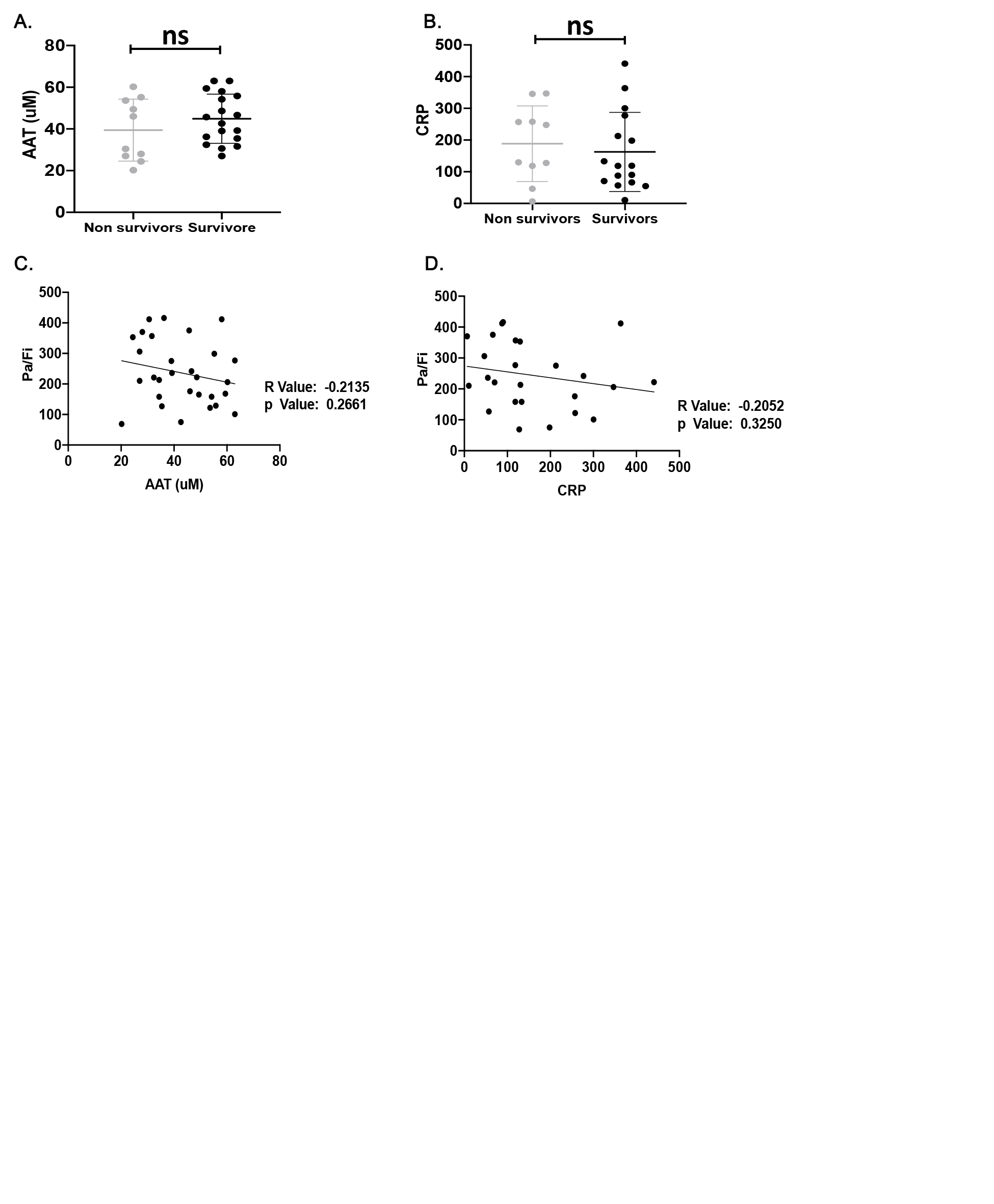

Supplement: S1 Fig — (A) Plasma AAT and (B) CRP levels in survivors compared to non-survivors. (C) Correlations between PaO2/FiO2 levels and plasma AAT, and (D) plasma levels of CRP. ns p>0.05 (two-tailed Mann-Whitney U test, Pearson correlation coefficients). (TIF) [file pone.0274427.s001.tif]
