# Supplementary material for: Correlation of alpha-1 antitrypsin levels and exosome associated neutrophil elastase endothelial injury in subjects with SARS-CoV2 infection
Source: PLoS One. 2022 Sep 9;17(9):e0274427. doi: 10.1371/journal.pone.0274427 (PMC9462798; doi:10.1371/journal.pone.0274427)
Supplement: S1 File — (DOCX) [file pone.0274427.s003.docx]

These two blots are one top and bottom pieces of one single membrane.


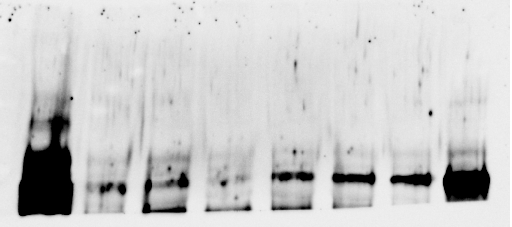


CD66 blot, Figure 3B


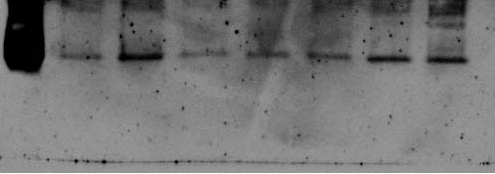


NE blot, Figure 3B


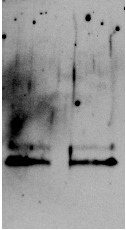


CD66, Figure 5D


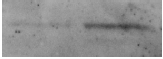


CD81, Figure 5D

Since the signal for CD81 was too low, unfortunately we had to crop the picture in the developer software to make it a little darker. So, the software exported the SCN file to JPG as it was cropped .
